# Impairment of carbonic anhydrase IX ectodomain cleavage reinforces tumorigenic and metastatic phenotype of cancer cells

**DOI:** 10.1038/s41416-020-0804-z

**Published:** 2020-03-25

**Authors:** Ivana Kajanova, Miriam Zatovicova, Lenka Jelenska, Olga Sedlakova, Monika Barathova, Lucia Csaderova, Michaela Debreova, Lubomira Lukacikova, Katarina Grossmannova, Martina Labudova, Tereza Golias, Eliska Svastova, Andreas Ludwig, Petr Muller, Borivoj Vojtesek, Jaromir Pastorek, Silvia Pastorekova

**Affiliations:** 1 Department of Tumor Biology, Institute of Virology, Biomedical Research Center, Slovak Academy of Sciences, Dubravska cesta 9, 84505 Bratislava, Slovakia; 2 Institute of Pharmacology and Toxicology, RWTH Aachen University, Wendlingweg 2, 52074 Aachen, Germany; 3 RECAMO, Masaryk Memorial Cancer Institute, Zluty kopec 7, 65653 Brno, Czech Republic

**Keywords:** Cancer microenvironment, Mechanisms of disease

## Abstract

**Background:**

Carbonic anhydrase IX (CA IX) is a hypoxia-induced enzyme regulating tumour pH and facilitating cell migration/invasion. It is primarily expressed as a transmembrane cell-surface protein, but its ectodomain can be shed by ADAM17 to extracellular space. This study aims to elucidate the impact of CA IX shedding on cancer cells.

**Methods:**

We generated a non-shed CA IX mutant by deletion of amino acids 393–402 from the stalk region and studied its phenotypic effects compared to full-length, shedding-competent CA IX using a range of assays based on immunodetection, confocal microscopy, in vitro real-time cell monitoring and in vivo tumour cell inoculation using xenografted NMRI and C57BL/6J female mice.

**Results:**

We demonstrated that the impairment of shedding does not alter the ability of CA IX to bind ADAM17, internalise, form oligomers and regulate pH, but induces cancer-promoting changes in extracellular proteome. Moreover, it affects intrinsic properties of cells expressing the non-shed variant, in terms of their increased ability to migrate, generate primary tumours and form metastatic lesions in lungs.

**Conclusions:**

Our results show that the ectodomain shedding controls pro-tumorigenic and pro-metastatic roles of the cell-associated CA IX and suggest that this phenomenon should be considered when developing CA IX-targeted therapeutic strategies.

## Background

Ectodomain cleavage (also called shedding) is an important regulatory phenomenon that controls abundance and/or function of cell-membrane-bound proteins as well as molecular composition of the extracellular microenvironment and thereby influences both cell’s phenotype and intercellular signalling. Deregulation of this process leading to imbalance between cell-associated and soluble variants of certain proteins has been associated with diverse diseases including infections, inflammatory disorders and cancer.

Ectodomain cleavage selectively occurs in only a small percentage of cell-surface proteins, such as receptors (Notch, IL6-R, VEGFR2, IGF2R), adhesion molecules (E-cadherin, CD44), growth factors (HB-EGF, TNFα, NRG1, Jagged), enzymes (ACE) and other molecules.^1,2^ Shedding of these molecules is generally low under basal conditions, but can be considerably induced by phorbol esters, ionophores, growth factors, cytokines, stress factors, cytotoxic drugs and other modulators of intracellular signalling that work via activation of enzymes executing the cleavage. Most transmembrane proteins are shed by metalloproteinases and/or proteases belonging to the disintegrin and metalloproteinase (ADAM) family. Prototypic member of this family ADAM17 (initially named TNFα converting enzyme, TACE) is currently known to cleave around 80 biologically relevant transmembrane proteins.^2,3^

Expression and activity of ADAM17 is induced by hypoxia, a typical feature of many solid tumours occurring due to poor and irregular blood flow caused by aberrant tumour vasculature.^4^ Cancer cells adapt to this microenvironmental stress by reprogramming their metabolism, proliferation, adhesion, migration, invasion and other features that allow them to survive, progress to metastasis and acquire therapy resistance.^5^ These adaptations are primarily mediated by molecular mechanisms driven by the hypoxia-inducible factor (HIF), which transactivates numerous genes including carbonic anhydrase IX.^6^

Carbonic anhydrase IX (CA IX) is a well-established biomarker of hypoxia, which exists as a type I transmembrane protein localised on the surface of tumour cells as well as a shed extracellular ectodomain, circulating in the blood of tumour patients.^7,8^ Cell-associated CA IX is present in a broad range of tumours both in cancer and stromal cells, and is associated with an aggressive tumour phenotype.^9^ Recent meta-analysis of the publicly available clinical data confirms that patients with high CA IX expression in tumours have higher risk of locoregional failure, disease progression, and higher risk to develop metastases, independent of tumour type or site.^10^ On the other hand, biological and clinical significance of the circulating CA IX ectodomain remains unclear, albeit several papers demonstrate its potential as a non-invasive cancer biomarker (reviewed in ref. ^9^).

Hypoxia induces CA IX expression via HIF1-mediated transcriptional activation. In addition, hypoxia regulates correct splicing of the CA IX transcript, and functionally activates the extracellular portion of the protein via PKA-mediated phosphorylation of its intracellular part.^6,11,12^

CA IX is a highly active enzyme belonging to the family of carbonic anhydrases that catalyse the inter-conversion between carbon dioxide and bicarbonate plus proton and are vital for ion transport and acid-base balance in virtually all organisms.^13^ Out of 12 catalytically active human isoforms, CA IX is the only isoenzyme that is predominantly associated with tumours and rarely expressed in differentiated healthy tissues. The role of CA IX in tumour cells is connected mostly to its enzyme activity, which is implicated in two interrelated processes contributing to tumour phenotype, namely pH regulation and cell migration/invasion.^14,15^ CA IX facilitates both acidification of the extracellular pHe and alkalisation of the intracellular pHi by a mechanism that involves catalysis of pericellular CO_2_ hydration, extracellular accumulation of protons and import of bicarbonate ions into the cytosol by bicarbonate transporters, such as NBC1 and AE2.^16–19^ Moreover, CA IX can use a non-catalytic mechanism to accelerate the export of lactate and protons by monocarboxylate transporters and further support acidic pHe and alkaline pHi.^20^ Slightly alkaline pHi provides survival and/or proliferative advantage by enabling biosynthetic reactions, whereas acidic pHe induces cell migration and activates proteases degrading the extracellular matrix, thereby supporting the invasion of tumour cells into the surrounding tissue.^21^ Thus, CA IX protects tumour cells from hypoxia as well as from acidosis, which develops due to the excess of lactate, protons and CO_2_ generated by oncogenic metabolism.^22,23^ Moreover, CA IX facilitates cell migration and invasion that enable the cells to escape from the hostile hypoxic and/or acidic microenvironment.^24–28^ Additional studies also demonstrate that CA IX endows tumour cells with stem-like properties and therapy resistance.^29^

However, CA IX is not only a HIF1 target and an effector of adaptation to hypoxia, but also a substrate of ADAM17, which cleaves its ectodomain at low constitutive rate that can be massively elevated in response to cytotoxic compounds including chemotherapeutic drugs.^7,30^

This study was undertaken with the aim to get insight into the impact of CA IX ectodomain cleavage on phenotype of tumour cells. To this end, we generated a non-shed (NS) deletion mutant of CA IX and studied biological consequences of its expression in comparison to the full-length CA IX protein, which is shedding-competent. We show that the impairment of shedding has profound effects on intrinsic properties of cells expressing the NS variant, in terms of their altered secretome, increased migration capacity, as well as improved ability of tumour growth and metastatic colonisation of lungs in xenografted mice that were used as a model of human cancer development in vivo.

The data presented here support the view that shedding of the CA IX ectodomain is a biologically relevant process that should be taken into account when attributing functions to cell-associated CA IX and when developing new CA IX-targeted therapeutic strategies.

## Methods

### Cell culture and transfection

Experiments described in this paper were performed with C33a human cervical cancer cells, MDCK canine kidney cells, and B16 F0 mouse melanoma cells transfected with the full-length CA9 cDNA (C33a-FL-CA IX; MDCK-FL-CA IX; B16-FL-CA IX) and del393–402 CA9 cDNA (C33a-NS-CA IX; MDCK-NS-CA IX; B16-NS-CA IX) in pcDNA3.1+ plasmid. Related mock-transfected cells served as negative controls. Wild-type CHO-wt hamster ovary cells, their ADAM17- defective derivative CHO-M2 cells and CHO-M2-TACE cells were kindly provided by Joaquin Arribas.^3^ The CHO cells were transiently transfected with the full-length human CA9 cDNA and its deletion mutants del393–396, and del393–402 generated by an inverse PCR using Fusion High-Fidelity DNA Polymerase (Thermo Fisher Scientific, MA, USA). The cells were routinely cultivated in DMEM medium with 10% FCS (BioWhittaker, Lonza, Basel Switzerland) at 37 °C in 5% CO_2_ in air. Hypoxic incubations were performed in an anaerobic workstation (Ruskinn Technologies, Bridgend, UK) in a humidified atmosphere containing 2% O_2_ (or 0.5% O_2_), 5% CO_2_, 10% H_2_ and 83% N_2_ (or 84.5% N_2_) at 37 °C. Transfections were performed using TurboFectTM transfection reagent (Thermo Fisher Scientific, MA, USA). To obtain stable polyclonal cell lines, transfected cells were subjected to selection in G418 for 2 weeks and then separated on magnetic beads (Dynabeads M-450 Tosylactivated, Invitrogen, CA, USA) coupled to CA IX-specific M75 antibody^31,32^ according to manufacturer´s instructions. Separated cell subpopulations were expanded and CA IX expression was analysed by flow cytometry, western blotting and immunofluorescence. Silencing of ADAM17 in C33a cells was performed by transduction of ADAM17 shRNA-expressing lentiviruses produced by transient transfection of HEK293T packaging cells according to standard protocols. In brief, sub-confluent HEK293T cells were co-transfected with 10 µg of the pLVTHM-hADAM17-2061 plasmid,^33^ 6 µg of pPAX2, and 6 µg of pMD2.G-VSVG using calcium phosphate precipitation. The medium was changed after 6 h, and the lentivirus containing supernatants were harvested after another 24 h and 48 h of incubation. To transduce C33a-FL-CA IX and C33a-NS-CA IX cells, 5 × 10^5^ cells were seeded into six wells and after 6 h, PEG precipitated virus particles and 10 µM polybrene were added. After 48 h, the cells were transduced for a second time. Transduced cells were sorted on BD FACSAriaTM (BD Biosciences) cell sorter based on EGFP produced by pVTHM lentivirus vector. Transduction efficiency of LsiADAM17 was 88% for C33a-FL cells and 81% for C33a-NS cells. Silencing of ADAM17 to about 50% of original levels was confirmed by Western blotting.

### Cloning and purification of the recombinant FL-CA IX-SBP protein

The Gateway® cloning system (Invitrogen, CA, USA) was used to create pcDNA3-RBS-CA9-(1–459)-C-SBP-GWc plasmid encoding the full-length form of the CA IX protein with a C-terminally localised SBP-tag. HEK-FL-CA IX-C-SBP cells were stably transfected with the plasmid and cultured to confluence. Subsequently, cell pellets were lysed in RIPA buffer supplemented with protease inhibitors and avidin (Sigma-Aldrich, MO, USA) was added to final concentration of 10 µg/ml to block endogenous biotin. After 5 min incubation at 4 °C, lysates were mixed with the Pierce™ High-Capacity Streptavidin Agarose beads (Thermo Fisher Scientific, MA, USA) and incubated overnight at 4 °C on rotary stirrer. The beads binding the FL-CA IX-SBP protein were washed and subjected to cleavage with recombinant human rhADAM17.

### rhADAM17 peptide-substrate and CA IX cleavage activity assay

Catalytic activity of the rhADAM17 (100 ng/ml, purchased from R&D Systems, MN, USA) was measured by hydrolytic processing of *Fluorogenic Peptide Substrate III Mca-P-L-A-Q-A-V-Dpa-R-S-S-S-R-NH2* (20 µM, R&D Systems, MN, USA) followed by monitoring the increasing fluorescence intensity at excitation and emission wavelengths of 320 nm and 405 nm (top read), respectively, in kinetic mode for 10, 30 and 120 min at 37 °C with a Synergy H4 plate reader (BioTek, VT, USA). All reactions were performed in a fluorogenic buffer containing 25 mM Tris, pH 9.5, 150 mM NaCl, 2.5 µM ZnCL_2_ and 0.005% Brij-35. For the rhADAM17 in vitro CA IX cleavage activity evaluation, the FL-CA IX-C-SBP protein was immobilised to High-Capacity Streptavidin Agarose (Thermo Fisher Scientific, MA, USA), washed and incubated with recombinant ADAM17 (dissolved at a concentration of 100 ng/ml in 25 mM Tris, pH 9.5, containing 2.5 µM ZnCL_2_ and 0.005% Brij) for 3 h at 37 °C. Thereafter, the supernatant was analysed by ELISA as described below.

### CA IX shedding inhibition

The effect of ADAM10/17 inhibitor GI254023X^34^ on the release of soluble CA IX was evaluated by ELISA assay. Briefly, 2 × 10^5^ CHO-M2 and CHO-M2-TACE cells with defective and overexpressed human TACE/ADAM17, respectively, and C33a cells were transiently transfected with the full-length human CA9 cDNA and allowed to grow in 2 ml DMEM media supplemented with 10% FCS in 3.5 cm plates. After 48 h, media were aspirated and inhibitor GI254023X (100 μM) was added in serum-free media for 4 h at 37 °C with 5% CO_2_. Subsequently, 100 μl of diluted supernatant was analysed in ELISA as described below. Calibration was done using standard ranging from 0 to 2000 pg/ml.

### Immunofluorescence assay

Cells grown on glass coverslips were gently washed with PBS and fixed in ice-cold methanol at −20 °C for 5 min. Nonspecific binding was blocked by incubation with PBS containing 1% BSA for 30 min at 37 °C. Cells were then incubated with M75 antibody (5 μg/ml) diluted in hybridoma medium for 1 h at 37 °C followed by an anti-mouse Alexa Fluor® 488-conjugated antibody (Invitrogen, CA, USA) diluted 1:1000 in the blocking buffer for 1 h at 37 °C. The nuclei were stained with DAPI (Sigma-Aldrich, MO, USA). Finally, the coverslips were mounted onto slides in the Fluorescent Mounting Media (Sigma-Aldrich, MO, USA), and analysed by the confocal laser scanning microscope Zeiss LSM 510 Meta.

### Internalisation assay

C33a-FL-CA IX and C33a-NS-CA IX cells (300,000 cells per Petri dish) were plated on glass coverslips 24 h before the experiment. The live cells were incubated with the antibody VII/20 (50 μg/ml) diluted in culture medium at 4 °C for 30 min to recruit the MAb to CA IX on the cell surface. Subsequently, the cells were washed to remove any unbound antibody and transferred to 37 °C for 3 h to induce internalisation, or fixed in ice-cold methanol at −20 °C for 5 min. At the end of the 3 h treatment period, the cells were washed and fixed. After blocking and incubation with anti-ADAM17 rabbit polyclonal antibody (Santa Cruz Biotechnology, TX, USA, 1:100 in 1% BSA), the primary antibodies were visualised using mix of anti-mouse Alexa Fluor® 488 and anti-rabbit Alexa Fluor® 555 secondary antibodies (Invitrogen, CA, USA, 1:1000 in 1% BSA). Finally, the cells were mounted onto slides and analysed by the confocal laser scanning microscope Zeiss LSM 510 Meta.

### Enzyme-linked immunosorbent assay (ELISA)

Detection of CA IX in cell extracts or culture media was performed by sandwich ELISA using V/10 capture monoclonal antibody (10 µg/ml) specific for the CA domain and a mixture of biotinylated M75 and IV/18 detector antibodies (200 ng/ml) specific for the PG domain of CA IX as described previously.^7^ For activation of shedding, cells were treated with 20 µM phorbol-12-myristate-13-acetate (Sigma-Aldrich, MO, USA) for 3 h. Detection of CA IX in serum samples from tumour-implanted mice was done by commercially available Human Carbonic Anhydrase IX DuoSet ELISA (R&D Systems, MN, USA).

### Western blotting

Western blotting was performed as described earlier.^7^ Membranes were probed with the following antibodies: M75 hybridoma medium (1:3 in 5% non-fat dry milk with 0.2% Nonidet P40 in PBS, 1 h, RT); anti-β-actin (Cell Signalling, 1:5000 in 3% BSA in TBS-T buffer, 1 h, RT); anti-ADAM17 (Santa Cruz Biotechnology, TX, USA, 1:300 in 3% BSA in TBS-T buffer, 2 h, RT); anti-IGFBP-3 (R&D Systems, MN, USA, 1:500 in 3% BSA in TBS-T buffer, overnight, 4 °C); anti-IGFBP2 (GeneTex, CA, USA, 1:1000 in 3% BSA in TBS-T buffer, overnight, 4 °C), anti-THBS-1 (Thermo Fisher Scientific, MA, USA, 1.5 µg/ml in 3% BSA in TBS-T buffer, 1 h, RT); anti-EMMPRIN/Basigin (Invitrogen, CA, USA, 1:250 in 3% BSA in TBS-T buffer, 2 h, RT); anti-mouse-HRP or anti-rabbit-HRP (Sigma-Aldrich, MO, USA, 1:5000 in 5% non-fat dry milk with 0.2% Nonidet P40/0.1% Tween20 in PBS, 1 h, RT). Protein bands were detected using enhanced chemiluminescence kit (GE Healthcare Bio-Sciences, IL, USA).

### Measurement of extracellular pH

Cells were either maintained in normoxia (21% O_2_) or exposed to hypoxia (2% O_2_) for 48 h. pH of cell culture media was measured by a microelectrode (InLab® Micro, Mettler Toledo, OH, USA) designed for small volumes. Values of extracellular pH in cells grown in constant medium volumes were obtained in four independent experiments with three parallel dishes for each cell line.

### Cell dissociation assay

For cell dissociation assay, MDCK cells were grown to high density, washed twice with PBS containing 2 mM CaCl_2_ and 2 mM MgCl_2_, detached using a rubber policeman and passed 10 times through a 1 ml micropipette. Number of disrupted particles (Np) was then count using Z2 Coulter (Beckman Coulter, CA, USA). Total number of cells (Nc) was obtained by counting the cells from the parallel monolayer fully dissociated by trypsinisation. The extent of dissociation was expressed as a ratio of Np/Nc. Data were obtained in two independent experiments with 6 parallel measurements for each cell type.

### Wound-healing assay

Cells were seeded to confluence in 12-well tissue culture plates, allowed to adhere and then starved overnight in DMEM with 0.5% FCS and wounded with a sterile micropipette tip. Floating cells were removed by washing with PBS. Fresh 0.5% FCS DMEM was then added. Time-lapse acquisition was performed by Zeiss Cell Observer System (Carl Zeiss AG, Oberkochen, Germany) at magnification ×100, in the incubation chamber at 37 °C in 21% O_2_ and 5% CO_2_ atmosphere. Imaging was done by Axiovision 4.8 software, using the Multidimensional Acquisition settings. Movement of the migrating fronts of cells covering the wound was evaluated for at least 15 positions in each sample. Positions were selected at a sufficient distance from each other and their fields of view never overlapped. Wound healing was quantified using ImageJ software as the wound area covered by cells in 24 h and 48 h.

### Real-time monitoring of migration and invasion with xCELLigence system

The xCELLigence cell index impedance measurements were performed using the CIM-Plate16 placed in the RTCA DP station according to the instructions of the supplier (Roche, Basel, Switzerland). Cells were trypsinised, resuspended at the density of 400,000 cell/ml in serum-free medium, added to the top chamber of the CIM-Plate and allowed to migrate towards bottom chamber containing medium with 10% FCS as a chemoattractant. The CIM-Plate 16 was placed in the RTCA DP station and migration was monitored every 15 min for 100 h. Invasion assay was done similarly using the Matrigel coating in the top chamber.

### Proximity ligation assay

Proximity ligation assay (PLA) was performed in a humid chamber at 37 °C according to the manufacturer’s instructions (Olink Bioscience, Uppsala, Sweden). Cells were seeded on glass coverslips and allowed to attach before transfer to 0.5% O_2_ atmosphere for 48 h. Then they were fixed with methanol, blocked with 3% BSA/PBS for 30 min, incubated with a mixture of above-described antibodies against CA IX and ADAM17 or AE2 (GenScript, NJ, USA) for 1 h, incubated with plus and minus PLA probes for 1 h, then incubated with ligation mixture containing connector oligonucleotides for 30 min, and finally with amplification mixture containing fluorescently labelled DNA probe for 100 min. The samples were analysed using a Zeiss LSM 510 Meta confocal microscope.

### Proteome profiler array analyses

PPA analyses were performed using the Human XL Cytokine Array Kit and Human Soluble Receptor Array Non-Hematopoietic panel kit (R&D Systems, MN, USA). Briefly, analysed C33a cell lines were seeded on cell culture dishes at a density 45 000 cells/cm^2^ and incubated for 24 h as above. Culture medium was replaced with serum-free DMEM with defined volume and cultures were cultivated for 48 h in normoxic or in hypoxic (2% O_2_) conditions. Conditioned media were then collected and centrifuged at 1000 rpm for 5 min to remove debris. Five independent samples of conditioned media for each cell line were mixed and allowed to interact on membranes with spotted antibody arrays overnight at 4 °C. Proteins were quantified by measuring the accumulated pixel density of the individual spots and adjusted based on reference spots using the ImageJ software.

### Quantitative PCR (qPCR)

Total RNA was isolated using RNeasy Plus Mini Kit (Qiagen, Hilden, Germany) and reverse transcription of 1 μg RNA for each sample was performed with the High-Capacity cDNA Reverse Transcription kit (Applied Biosystems, CA, USA). qPCR was carried out using Maxima Syber Green PCR Master mix (Thermo Fisher Scientific, MA, USA), gene-specific primers listed below and ran for 10 min at 95 °C for initial denaturation followed by 40 cycles of 95 °C for 15 s and 60 °C for 1 min. Sample CT values were normalised to β-actin. Relative expression was calculated using the ΔΔCT method. All amplifications were performed in triplicates. Results were calculated from two independent experiments. The oligonucleotides used for qPCRs were as follows—*ADAM17* sense: 5′-TGGATGAAGGAGAAGAGTGTGA-3′ and antisense: 5′-AAGATCCAAGCAAACAGTGTCAT-3′, *IGFBP-3* sense: 5′- CAGAGACTCGAGCACAGCAC-3′ and antisense: 5′- GATGACCGGGGTTTAAAGGT-3′, *IGFBP-2* sense: 5′- GACAATGGCGATGACCACTCA-3′ and antisense: 5′- GCTCCTTCATACCCGACTTGA-3′, *IGF1* sense: 5′- CTCTTCAGTTCGTGTGTGGAGAC-3′ and antisense: 5′- CAGCCTCCTTAGATCACAGCTC -3′, *β-actin* sense: 5′-TCCTCCCTGGAGAAGAGCTA-3′ and antisense: 5′-ACATCTGCTGGAAGGTGGAC-3′.

### In vivo experiments

NMRI-Foxn1^nu^ nu/nu female mice and C57BL/6J female mice (Charles River Laboratories, Inc.) were housed in SPF facility and used in accordance with the Institutional Ethic Committee guidelines under the approved protocols. The project was approved by the national competence authority—State Veterinary and Food Administration of the Slovak Republic (No. Ro. 4245/13-221 and 292/16–221 g) in compliance with the Directive 2010/63/EU and the Regulation 377/2012 on the protection of animals used for scientific purposes. Mice were housed in groups of 3 randomised animals in individually ventilated IVC (Tecniplast) cages with wooden fibres’ bedding, at 20 ± 2 °C temperature, using natural light/dark cycle, with SNIFF diet, ad libitum access to food and water, with environmental enrichment by paper houses. The mice were subjected to regular monitoring with humane endpoint. The number of mice were kept at minimum required to achieve statistical significance, in vivo study was designed based on thorough preceding in vitro experiments. Primary tumours were generated by a subcutaneous injection of a suspension of either B16-FL-CA IX or B16-NS-CA IX melanoma cells (1 × 10^6^ cells in 100 µl PBS), into the both right and left upper flank of NMRI-Foxn1^nu^ nu/nu female mice (*n* = 6 per each of two experimental groups corresponding to either FL or NS model, 5 weeks old, average weight 25±4 g). Tumour volumes were measured on the 7th, 11th and 14th day with digital calliper in home cages. On the day 14, mice were subjected to euthanasia by thiopental overdose and blood was acquired from the jugular vein immediately after the onset of anaesthesia. For the lung colonisation assay, C57BL/6J mice (*n* = 7 per each of three experimental groups, 4 weeks old, average weight 16.3 ± 3 g) were injected with 1.5 × 10^3^ B16-FL-CA IX/ B16-NS-CA IX melanoma cells in 200 µl PBS through the tail vein, control group consisted of three mice inoculated with PBS only. Mice were euthanised by thiopental overdose on the 21st day after tumour inoculation. Lungs from supine anesthetised mice were fixed via vascular perfusion using PBS and formalin. Lung area covered by tumour focuses was measured using ImageJ software.

### Melanin bleaching and immunohistochemistry

Dissected tumours were formalin-fixed and embedded in paraffin according to the standard histological procedure. Because of melanin presence in tissues, sections were bleached using potassium permanganate solution (2 g/l) for 20 min at 65 °C. After rinsing in PBS, sections were immersed in 1% oxalic acid for 1 min at room temperature followed by rinsing in PBS for 10 min. Immunohistochemistry was performed on Dako Autostainer employing DakoCytomation EnVision®+ System-HRP (DAB) for use with Mouse Primary Antibodies according to the manufacturer’s instructions. The sections were labelled with M75 antibody in hybridoma medium diluted 1:100 for 1 h at room temperature and after washing incubated with secondary anti-mouse-HRP antibody for 30 min at room temperature. Absence of nonspecific binding of the secondary antibody was confirmed on parallel sections by omitting the primary antibody. Staining was visualised using 3,3′-diaminobenzidine (DAB) as a chromogenic substrate for 1 min. The sections were counterstained with Mayer’s haematoxylin and mounted in Aquamount (Merck Millipore, MA, USA). The stained sections were examined using a Leica DM4500B microscope and images were captured by a Leica DFC480 camera.

### Statistical analysis

The significance of differences of pooled results were estimated by unpaired two-tailed Student’s *t*-test. *P* < 0.05 was assumed to be significant (*P < 0.05, **P < 0.01, ***P < 0.005). Statistical analysis was performed in GraphPad Prism (GraphPad Software).

## Results

### Deletions in the CA IX stalk region define the ADAM17 cleavage region and generate non-shed CA IX variant

**Fig. 1:**
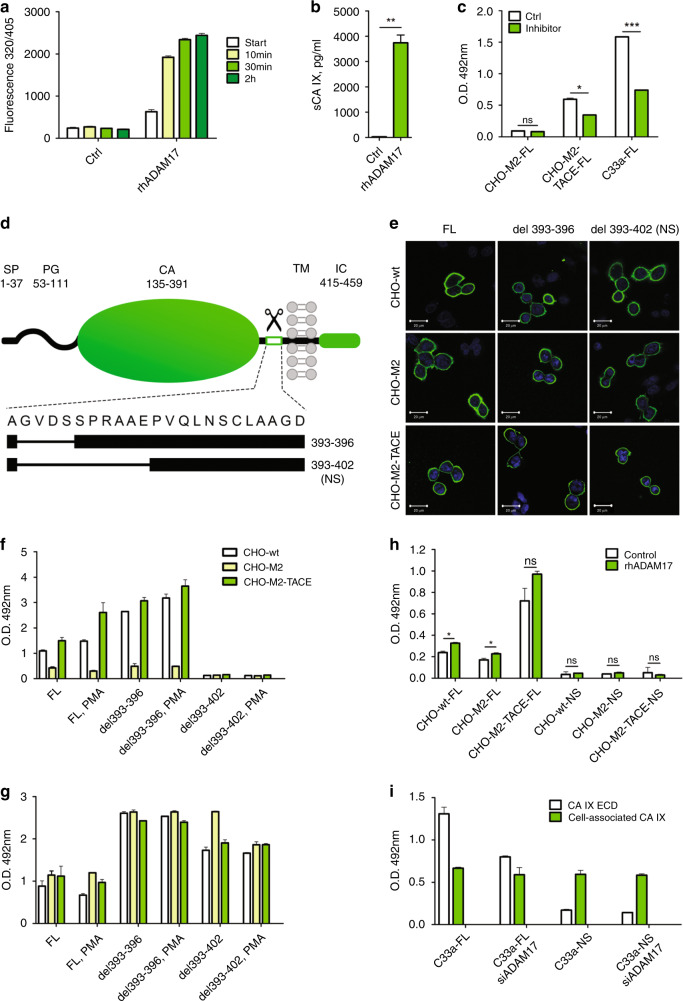
ADAM17 cleaves the CA IX ectodomain and deletion of amino acids 393–402 in the CA IX stalk region prevents the ECD shedding. **a** Verification of the cleavage activity of the recombinant human TACE/ADAM17 (rhADAM17) towards the fluorogenic peptide Mca-Pro-Leu-Ala-Gln-Ala-Val-Dpa-Arg-Ser-Ser-Ser-Arg-NH2. The peptide was used at the final concentration of 10 μM in a total of 100 μL reaction mixture with 10 ng of the rhADAM17. Time-related increase of the fluorescence emitted from the pro-TNF-α-derived peptide proves that rhADAM17 was active. **b** Recombinant CA IX-SBP fusion protein attached to a streptavidin-sepharose matrix was cleaved by rhADAM17, eluted and detected by ELISA using CA IX-specific monoclonal antibodies. **c** Cells transiently expressing FL-CA IX were treated with ADAM17/10 inhibitor for 24 h and CA IX ECD was detected in culture media by ELISA. Results in **b** and **c** support the role of ADAM17 in cleavage of CA IX. **d** Schematic illustration of the CA IX domain structure and positions of deletions in the mutants. Scissors indicates extracellular domain cleavage region. Missing amino-acid residues of individual deletion variants are represented by numbers on the right side. SP signal peptide, PG proteoglycan-like domain, CA catalytic domain, TM transmembrane region, IC intracellular tail. **e** Immunofluorescence analysis of CHO-wt, CHO-M2 and CHO-M2-TACE cells transiently expressing the FL-CA IX and two stalk deletion variants. The cells were fixed with methanol, incubated with M75 antibody followed by ALEXA Fluor488-secondary antibody and nuclei were stained with DAPI. Deletion of the cleavage site did not affect the CA IX localisation. **f**, **g** ELISA analysis of the CA IX deletion variants for the ECD shedding. The plasmids encoding FL-CA IX and its two deletion mutants were transiently transfected to CHO-wt, CHO-M2 (ADAM17-defective) and CHO-M2-TACE (human ADAM17 expressing) cell lines. 48 h after transfection, the cells were cultivated for 3 h in equal medium volumes in the presence or absence of PMA. Undiluted conditioned media (**f**) and cell lysates diluted 1:10 (**g**) were collected and examined by ELISA using V/10 antibody as a capture and mixture of biotinylated MAbs M75 and IV/18 as a detector. **h** Biochemical evidence that ADAM17 can cleave FL-CA IX, but not the NS mutant was obtained by treatment of CHO cell variants expressing FL and NS, respectively, with recombinant rhADAM17 added to medium at a concentration of 50 μg/ml for 24 h. Collected media were analysed by ELISA. **i** ADAM17 suppression resulting from infection by lentiviruses expressing ADAM17-specific shRNA led to a decreased CA IX shedding from C33a-FL cells, but not from C33a-NS cells proving the involvement of ADAM17 in the CA IX ECD cleavage. The results (mean ± SD) represent the mean of two measurements in triplicates. (*P < 0.05, ***P < 0.005, ns non-significant).

We previously demonstrated that CA IX shedding depends on the presence of ADAM17.^7^ In this study, we first wanted to obtain biochemical evidence that CA IX is a direct substrate of ADAM17. For that purpose, we used a recombinant human ADAM17 (rhADAM17), activity of which was verified by the cleavage of a commercial fluorogenic peptide derived from pro-tumour necrosis factor-α (Fig. 1a). Then we performed the rhADAM17-mediated cleavage of a human recombinant CA IX-SBP fusion protein bound to a streptavidin-sepharose matrix. Released CA IX ectodomain (ECD) was detected in ELISA, which confirmed that CA IX is an ADAM17 substrate (Fig. 1b). Moreover, we checked whether a dual ADAM17/ADAM10 inhibitor GW280264X^34^ can reduce the CA IX ECD shedding from the full-length (FL) CA IX-transfected CHO cells (Fig. 1c), which do not contain any endogenous CA IX (Suppl. Fig. 1). Notably, wild-type CHO cells express a functional hamster ADAM17, whereas their derivative CHO-M2 cells are defective in the surface hamster ADAM17 due to its impaired trafficking and activation that prevents the cleavage of its substrates.^35^ In CHO-M2-TACE cells, this defect is repaired by a stable ectopic expression of the functional human ADAM17/TACE. CHO-M2 cells and CHO-M2-TACE cells transiently expressing FL-CA IX were incubated for 24 h in the presence or absence of the ADAM17/10 inhibitor and their culture media were collected and analysed by sandwich ELISA (Fig. 1c). Results of this experiment showed that the CA IX ECD is shed from the CHO-M2-TACE cells expressing FL-CA IX and that the shedding can be significantly reduced by the ADAM17/10 inhibitor, with the rate of inhibition similar to the one observed earlier.^34^ On the other hand, the CA IX ECD shedding from the FL-CA IX-expressing CHO-M2 cells was negligible in accord with the defect in ADAM17 and no effect of the inhibitor was seen. However, significant reduction of CA IX shedding with the inhibitor was observed using C33a human cancer cells ectopically expressing FL-CA IX (Fig. 1c).

Extracellular part of the CA IX protein (i.e. ECD) consists of an N-terminal proteoglycan-like region (PG, aa 53–111) linked through a hinge (aa 112–134) to a large catalytic domain (CA, aa 135–391) sitting on a membrane-proximal stalk (aa 392–414). The ECD is anchored in the plasma membrane via a single transmembrane region (TM, aa 415–434) that is extended into a short intracellular tail (IC, aa 435–459). This arrangement, illustrated on Fig. 1d, is similar to the other type I transmembrane substrates of ADAM17. Although there is no consensus amino-acid sequence for the ADAM17 cleavage site, shedding usually occurs in the stalk region with the preference for certain amino acids at defined positions.^1,36^

In order to generate a non-cleavable CA IX variant, we performed in vitro mutagenesis of the stalk region covering the amino acids 392–414 and generated two stalk deletion mutants del393-396 and del393-402, see Fig. 1d. Plasmids encoding the full-length (FL) CA IX and its deletion mutants were transiently transfected to the CHO-wt cells, ADAM17-defective CHO-M2 cells and to CHO-M2-TACE cells expressing the human ADAM17. All three variants of the CA IX protein were correctly transported to the plasma membrane, as demonstrated by staining of transiently transfected CHO-wt, CHO-M2 and CHO-M2-TACE cells (Fig. 1e). Transfected cells were then incubated for 24 h in the presence and absence of phorbol myristate acetate (PMA) that is known as inducer of shedding and was also shown to activate the CA IX ECD cleavage.^7^ Culture media (Fig. 1f) and corresponding cell lysates (Fig. 1g) were harvested and levels of cleaved extracellular CA IX versus cell-associated CA IX were determined by ELISA. Figure 1g indicates that CA IX variants were expressed at different levels apparently as a result of different efficiency of transient transfection (see Suppl. Fig. 1). However, as clearly evident from Fig. 1f, deletion of the amino acids 393–396 affected neither the basal nor the PMA-induced CA IX ECD shedding in ADAM17-competent CHO-wt and CHO-M2-TACE cells. On the other hand, deletion of the amino acids 393–402 led to a full elimination of both basal and activated shedding of the CA IX ECD in both cell types. Therefore, the deletion variant del393-402 was designated NS (as “non-shed”). Expectedly, shedding did not occur in any variant of ADAM17-defective CHO-M2 cells. Addition of rhADAM17 to CHO cells led to the ECD cleavage only from FL, but not from NS variant of CA IX (Fig. 1h), supporting the view that the region spanning amino acids 397–402 contains the ADAM17 cleavage site. Finally, suppression of ADAM17 in C33a-FL cells transduced by lentiviruses expressing ADAM17-specific shRNA resulted in reduced CA IX ECD cleavage (Fig. 1i). This effect was not observed in the transduced C33a-NS cells, further strengthening our conclusions on the involvement of ADAM17 in the CA IX ECD cleavage as well as on the position of the cleavage site.

Perturbed shedding of the NS-CA IX mutant lacking the amino acids 393–402 could not be attributed to its lower availability to the ADAM17 proteinase, because it was properly localised on the cell surface, (Fig. 1e and Suppl. Fig. 1). Thus, the NS-CA IX deletion variant del393–402, which completely lost the ADAM17 cleavage site, was used for further experiments in comparison to FL-CA IX.

### NS-CA IX variant interacts with ADAM17 and retains typical features of FL-CA IX

Data from the literature indicate that the ADAM17-mediated ectodomain cleavage also requires sites distal from the cleavage site to recognise the substrate.^2^ Therefore, we decided to analyse whether the cleavage site deletion affected the recognition of the NS-CA IX variant by ADAM17. To this end, we generated stable C33a cell lines with the constitutive expression of the NS-CA IX and FL-CA IX proteins. To avoid possible bias of clonal differences, we used M75 antibody-mediated magnetic separation of the transfected CA IX-expressing cells from the background of the CA IX-negative cells. Thereby, we obtained mixed populations of transfectants that were expanded and in the course of the following experiments regularly checked for the CA IX expression by flow cytometry and/or western blotting (Suppl. Fig. 2a). Expression levels of ectopically expressed FL-CA IX and NS-CA IX in transfectants were similar to levels of the CA IX protein naturally expressed in various cancer cell lines in response to hypoxia (Suppl. Fig. 2b).

**Fig. 2:**
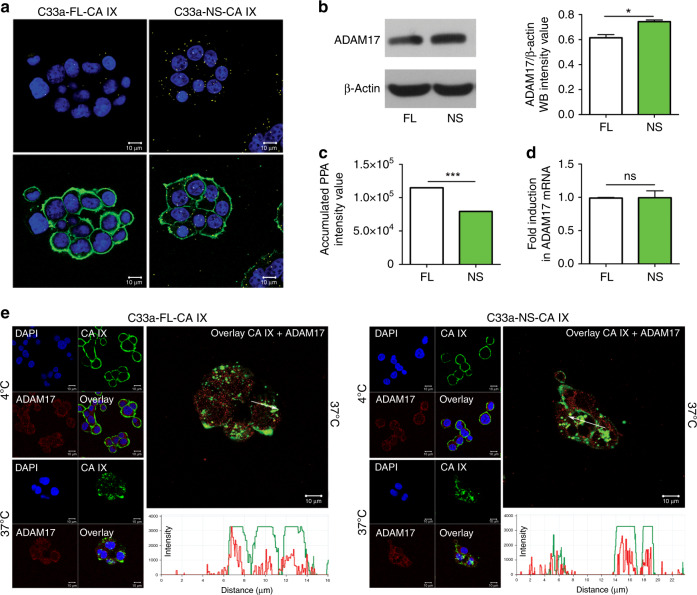
Relationship of FL-CA IX and NS-CA IX to ADAM17. **a** Proximity ligation assay (PLA) with CA IX-specific M75 MAb and anti-human ADAM17 polyclonal antibody performed in C33a-FL-CA IX and C33a-NS-CA IX cells plated on coverslips and fixed with methanol. Yellow PLA signal indicating the interaction of CA IX with ADAM17 was clearly visible and was much stronger in the cells expressing NS-CA IX than that in FL-CA IX cells. CA IX protein was post-labelled in green. **b** Western blotting analysis of the ADAM17 level in C33a-FL-CA IX and C33a-NS-CA IX cell extracts (left) with intensities of the protein bands evaluated by ImageJ. Data were obtained in two independent experiments. **c** Analysis of extracellular ADAM17 level using Proteome Profiler Human Array, Non-hematopoietic panel (ARY012, R&D Systems, MN, USA) probed with serum-free culture media collected from C33a-FL-CA IX and C33a-NS-CA IX cells. The graph shows intensities of ADAM17-specific spots. **d** Quantitative PCR analysis of relative ADAM17 mRNA level in C33a-FL-CA IX versus C33a-NS-CA IX cells normalised to the level of β-actin mRNA. Data were obtained in three independent experiments. **e** Colocalisation of membrane CA IX (4 °C) and intracellular/internalised CA IX (37 °C) with ADAM17 was analysed by immunofluorescence and confocal microscopy. Monolayers of C33a-FL-CA IX and C33a-NS-CA IX cells were fixed and double-stained for CA IX (green) and ADAM17 (magenta). Clear overlap of the two staining signals was evident at both CA IX variants. Experiments were repeated twice and performed in triplicates. (*P < 0.05, ***P < 0.005, ns non-significant).

To visualise the possible contact between the FL-CA IX and ADAM17 in comparison to the NS-CA IX and ADAM17, we used a proximity ligation assay (PLA) that allows for in situ detection of proteins, interaction of which brings them to close proximity.^37^ Using antibodies specific for CA IX and ADAM17, we detected the PLA signal in both C33a-FL-CA IX and C33a-NS-CA IX (Fig. 2a), but not in the control, CA IX-negative C33a cells (not shown). Similar results were obtained in transiently transfected CHO cells (Suppl. Fig. 3). Thus, the ADAM17 cannot cleave the NS-CA IX but can recognise it as a substrate and can bind to its extracellular part. Interestingly, the PLA signal was stronger in C33a cells expressing NS-CA IX than that in FL-CA IX cells. In accord, western blotting analysis of cell extracts revealed that the C33a-NS-CA IX cells contain a higher amount of the ADAM17 proteinase when compared to the C33a-FL-CA IX cells (Fig. 2b), while the extracellular ADAM17 levels were lower in the culture medium of NS-CA IX-expressing cells than in the medium of FL-CA IX cells as demonstrated by a proteome profiler array (Fig. 2c). qPCR did not reveal any significant differences in ADAM17 mRNA levels between NS-CA IX and FL-CA IX transfectants (Fig. 2d). This suggests that the increase in ADAM17 protein in the presence of NS-CA IX may be due to its stabilisation.

We then wanted to learn whether the NS-CA IX protein could internalise in response to antibody-induced endocytosis and whether the internalised NS-CA IX remained at least in part co-distributed with ADAM17. For that purpose, we used VII/20 monoclonal antibody that induces endocytosis of CA IX via binding to its catalytic domain exposed on the cell surface.^38^ Figure 2e shows that NS-CA IX is capable of internalisation comparably to FL-CA IX, and that the immunofluorescence signal generated by the CA IX-specific antibody in both cases overlaps with the intracellular signal from the ADAM17 antibody.

We further investigated, whether the NS-CA IX mutant retained typical features of FL-CA IX using the above-described C33a transfectants, as well as analogously-generated transfectants of MDCK cells. Western blotting analysis of cellular extracts from C33a-FL-CA IX cells and C33a-NS-CA IX cells showed that both CA IX protein variants were expressed at similar levels and could equally form oligomers in non-reducing conditions (Suppl. Fig. 4a). The same result was obtained in MDCK-NS-CA IX cells compared to MDCK-FL-CA IX cells. We also performed ELISA analysis of the ectodomain levels in culture media collected from C33a-FL-CA IX and C33a-NS-CA IX cells to be sure that NS-CA IX did not undergo shedding in C33a cells. The results presented in Suppl. Fig. 4b show that the medium collected from C33a-NS-CA IX cells did not contain the CA IX ectodomain irrespective of whether they were incubated in the absence or presence of PMA.

Moreover, we employed proximity ligation assay (PLA) to demonstrate that both FL-CA IX and NS-CA IX could interact with anion exchanger AE2, a bicarbonate transporter known to bind CA IX as demonstrated in earlier studies, see Suppl. Fig. 4c. These findings indicate that the pH-regulatory function of CA IX was not perturbed by the cleavage-disabling deletion in the CA IX stalk region. We also measured extracellular pH in the culture media of C33a-FL-CA IX and C33a-NS-CA IX cells immediately after 48 h incubation in 2% hypoxia. We found that the NS-CA IX variant was capable of acidifying the extracellular pH to the same extent as FL-CA IX (Suppl. Fig. 4d).

### NS-CA IX and FL-CA IX differentially affect the extracellular proteome

**Fig. 3:**
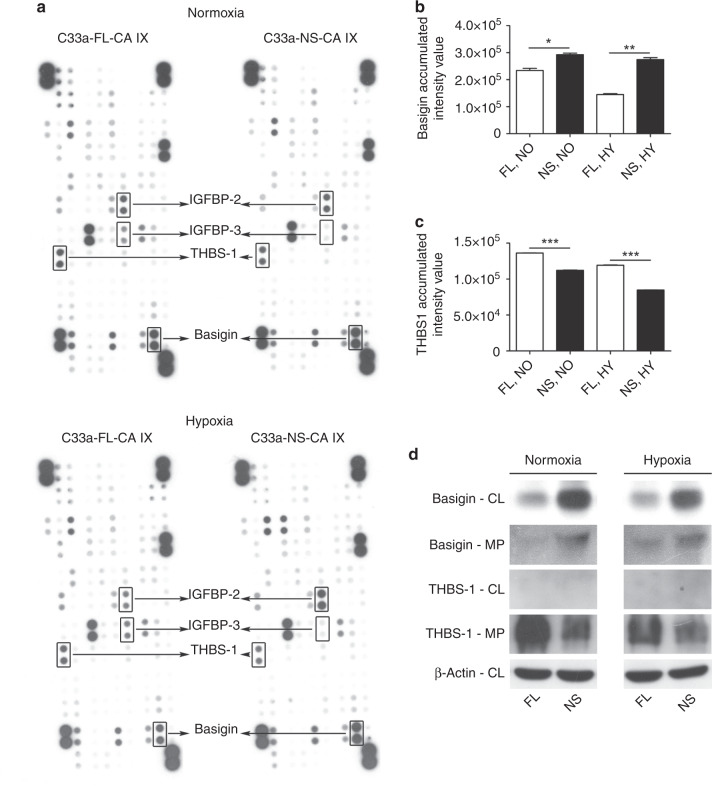
CA IX ectodomain cleavage affects the extracellular proteome of C33a cells in normoxia and hypoxia. **a** Proteome profiler Human XL Cytokine Array (ARY022, R&D Systems, MN, USA) probed with serum-free-conditioned medium (SF-CM) of C33a-FL-CA IX or C33a-NS-CA IX cells incubated under normoxic (NO) and hypoxic (HY) conditions for 48 h. Spot doublets of corresponding proteins with differential intensities are framed and indicated by arrows. Medium pooled from five independent experiments was used for the array. Spot intensities representing **b** pro-oncogenic basigin, and **c** anti-angiogenic thrombospondin-1 were analysed in culture medium samples of C33a-FL-CA IX versus C33a-NS-CA IX cells. Abundance of the proteins was assessed according to spot intensity using densitometry. Data are shown as mean ± SD. **d** Validation of the differential levels of basigin and thrombospondin-1, respectively, by Western blotting analysis of medium precipitates (MP) and cell lysates (CL) of normoxic (NO) and hypoxic (HY) cells. (*P < 0.05, **P < 0.01, ***P < 0.005).

We then investigated whether the NS-CA IX mutant could impact the extracellular proteome differently than the FL-CA IX protein using proteome profiler arrays of antibodies specific for a range of cytokines, growth factors and other regulatory proteins. Serum-free culture media collected from normoxic and hypoxic C33a-NS-CA IX and C33a-FL-CA IX cells, were overlaid on membranes with spotted antibody arrays and allowed to interact. Abundance of the bound proteins was then assessed according to intensity using densitometry. Results of the assay showed that NS-CA IX expression was associated with altered extracellular levels of several regulatory molecules (Fig. 3a), specifically with increased levels of pro-oncogenic basigin and decreased levels of anti-angiogenic thrombospondin-1 (THBS1). The changes were apparent both in normoxia and hypoxia, see Fig. 3a-d.

**Fig. 4:**
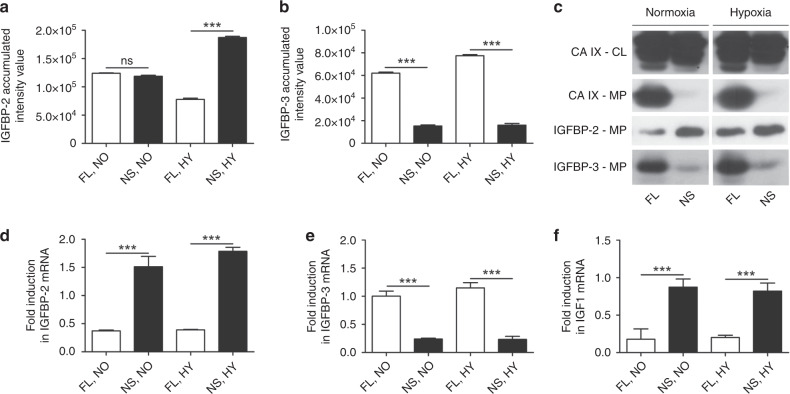
Increased IGFBP-2 and reduced IGFBP-3 in C33a-NS-CA IX cells. Analysis of the differential expression of **a** IGFBP-2 and **b** IGFBP-3 in SF-CM samples from C33a-FL-CA IX and C33a-NS-CA IX incubated under normoxic (NO) and hypoxic (HY) conditions for 48 h and evaluated by Proteome Profiler Human XL Cytokine Array (ARY022, R&D Systems, MN, USA). Abundance of the proteins was assessed according to spot intensity using densitometry. Data are shown as mean ± SD of five independent samples. **c** Western blotting analysis of CA IX, IGFBP-2 and IGFBP-3 protein levels in medium precipitates (MP) of C33a-FL-CA IX and C33a-NS-CA IX cells. Impairment of shedding in NS-CA IX is associated with increased IGFBP-2 and reduced IGFBP-3 secretion to culture medium. Quantitative PCR analysis of relative mRNA levels of **d** IGFBP-2, **e** IGFBP-3 and **f** IGF1 in C33a-FL-CA IX versus C33a-NS-CA IX cells normalised to β-actin mRNA. (***P < 0.005, ns non-significant).

Moreover, we observed contrasting expression pattern of IGF1-binding proteins IGFBP3 (reduced in NS-CA IX cells) and IGFBP2 (increased in NS-CA IX cells), see Fig. 3a and Fig. 4. This pattern was confirmed by western blotting (Fig. 4c) and also by qPCR, which also showed that the levels of IGF1 transcripts were decreased in cells expressing FL-CA IX (Fig. 4d-f). Thus, based on the C33a model it is possible that the impairment of the CA IX ectodomain cleavage may affect cancer-related pathways and contribute to tumour-promoting microenvironment, but this assumption needs further exploration.

### Cells expressing NS-CA IX exhibit reduced dissociation but increased migration and invasion compared to FL-CA IX cells

Previous studies have shown that CA IX protein weakens intercellular contacts, increases cell migration and thereby facilitates the first steps of the metastatic cascade.^16,25^ These capabilities of CA IX were shown to depend on the preserved integrity of the ectodomain that contains the catalytic domain. Therefore, we wanted to learn whether blocking of the ectodomain release by deletion of the cleavage site affected these CA IX functions. To this end, we performed dissociation assay, using MDCK cells constitutively expressing NS-CA IX and FL-CA IX (see the Suppl. Figs. 2 and 4). MDCK cells are particularly suitable for this purpose as they normally show tight E-cadherin-mediated cell–cell adhesion that can be perturbed by CA IX via uncoupling E-cadherin from the adhesion complex containing beta-catenin.^39^

**Fig. 5:**
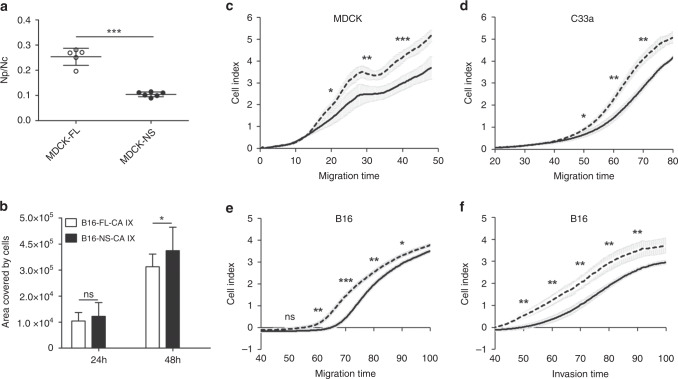
NS-CA IX variant decreases dissociation and increases cell migration and invasion compared to FL-CA IX. **a** Dissociation assay showed that MDCK-FL-CA IX monolayer was disrupted to significantly higher degree than MDCK-NS-CA IX cells, calculated as a ratio between the number of dissociated particles (Np) and a total number of cells (Nc). **b** In the wound-healing assay, B16-NS-CA IX cells exhibited faster migration compared to B16-FL-CA IX cells. The graph shows an area covered by migrating cells closing the wound at 24 h and 48 h after the scratch, measured at various positions along the wounds’ borders. Data from three independent experiments are shown as mean ± SD. **c, d, e** Cell migration was analysed using the xCELLigence Real-Time Cell Analyzer as described in Materials and Methods. MDCK, C33a and B16 cells (4 × 10^4^ /well) transfected with FL-CA IX (full line) and NS-CA IX (dashed line) were added in quadruplicates to the upper chambers of the CIM-Plate. Migration was expressed as cell index representing relative change of impedance monitored every 15 min for 100 h. **f** B16 cell invasion was analysed as in **e** using the upper chamber of the CIM plate covered with Matrigel. (*P < 0.05, ***P < 0.005, ns non-significant).

The dissociation assay is based on mechanical disruption of a confluent cell monolayer by several-fold pipetting, which generates single cells and/or cellular clusters. Number of the resulting particles is then correlated to the total number of enzymatically separated individual cells present in the same monolayer. Data from this assay show that NS-CA IX expression in MDCK cells led to a significantly reduced number of dissociated particles composed of more cells when compared to FL-CA IX, suggesting that the loss of the cleavage site decreases the ability of CA IX to perturb intercellular contacts (Fig. 5a).

In the next step, we investigated how cells expressing NS-CA IX versus FL-CA IX behave in terms of migration and invasion. Here we employed a B16 metastatic mouse melanoma cell model, which was also used for in vivo studies described below. Analogously to MDCK and C33a models, mouse B16 cells, which show negligible expression of the endogenous mouse CA IX (Suppl. Fig. 5) were permanently transfected with plasmids encoding human CA IX variants, subjected to magnetic separation, checked for expression of FL-CA IX and NS-CA IX, and expanded. First, we looked at the migration propensity of B16-NS-CA IX versus B16-FL-CA IX cells using a wound-healing assay. As shown in Fig. 5b, NS-CA IX makes B16 cells migrating significantly faster than FL-CA IX.

Furthermore, we tested the cells transfected with NS-CA IX versus FL-CA IX for proliferation, migration and/or invasiveness in real-time setting using the xCELLigence system, which measures the electrical impedance across gold microelectrodes integrated in the bottom of microplates as a result of changes in cell coverage.

MDCK and C33a cells expressing NS-CA IX and FL-CA IX, were analysed for migration (Fig. 5c,d), whereas B16-NS-CA IX and B16-FL-CA IX were analysed also for invasiveness (Fig. 5e,f). In all cases, there were no significant differences between NS-CA IX and FL-CA IX-expressing cells in proliferation (Suppl. Fig. 6). However, we observed a consistently and significantly increased migration and invasion of NS-CA IX-expressing cells, when compared to FL-CA IX transfectants (Fig. 5c–f).

### NS-CA IX expression is associated with increased tumour growth and metastasis compared to FL-CA IX

Next, we wanted to find out whether faster migration and invasion of the NS-CA IX expressing B16 cells were translated into better in vivo tumour growth and increased ability to form lung metastases in colonisation assay. The B16-FL-CA IX and B16-NS-CA IX cells, respectively, were each subcutaneously inoculated into both flanks of the mouse back (total 12 SPF, drug naïve animals in two groups per 6), and the tumours were allowed to grow for 2 weeks. After they became palpable, their diameters were measured in 3–4-day intervals by callipers, and the volume was calculated. At the end of the experiments, the mice were euthanised, serum samples were collected and the tumours were dissected and weighed. Sera of the non-inoculated mice were used for negative control. ELISA analysis confirmed that the sera of mice inoculated with B16-FL-CA IX contained 252 ± SD pg/ml of circulating CA IX, which roughly corresponds to the concentration found in human tumour patients.^40–43^ As expected, sera of mice inoculated with B16-NS-CA IX cells showed a diminished value of 28 ± SD pg/ml of CA IX, whereas sera of non-inoculated mice were CA IX-negative.

**Fig. 6:**
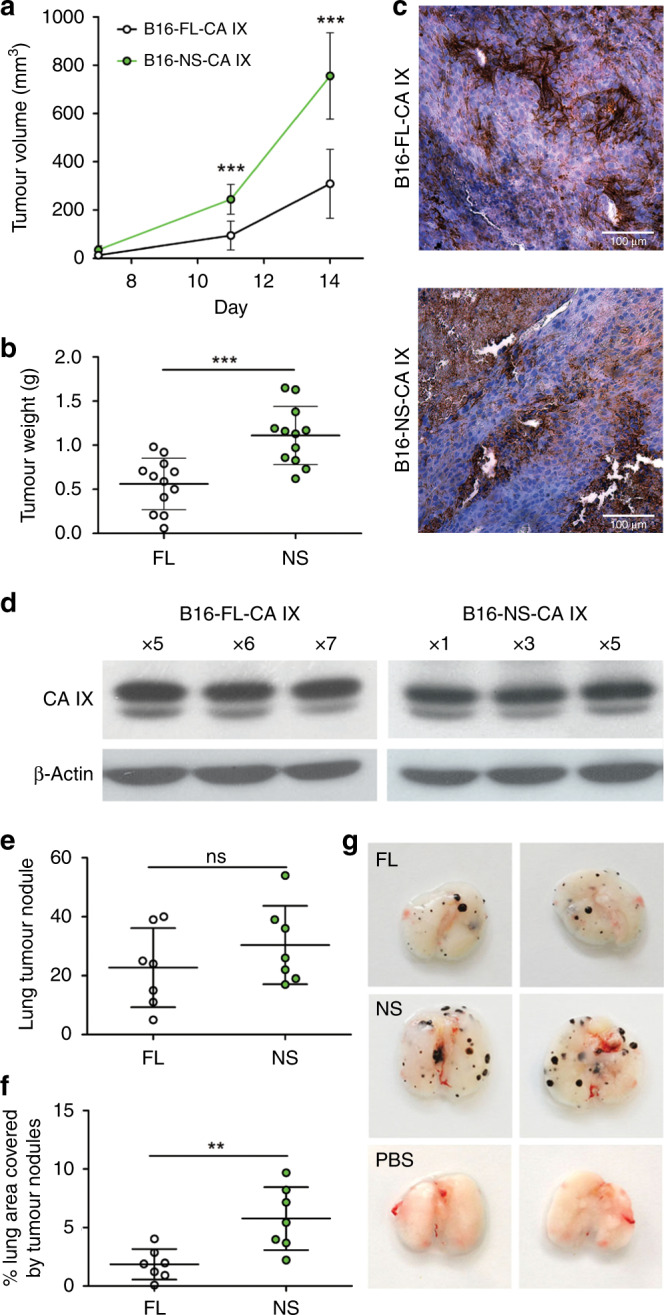
Impaired CA IX shedding results in increased tumour growth and lung colonisation in vivo. **a** Volumes and **b** weights of tumours grown from B16-FL-CA IX and B16-NS-CA IX cells implanted to NMRI-Foxn1^nu^ nu/nu mice (*n* = 6 for each group). Tumour volume was measured on the 7th, 11th and 14th day with digital calliper. Mice were sacrificed on 14 days after inoculation and tumours were weighed. **c** Immunohistochemistry demonstrating CA IX localisation in B16-FL-CA IX and B16-NS-CA-IX tumour tissues. Paraffin sections were stained with M75 antibody visualised with DAB, followed by haematoxylin counterstaining. **d** Western blotting analysis showing CA IX expression in B16-FL-CA IX and B16-NS-CA IX tumour homogenates of three different animals for each group (numbers shown above pictures). **e**, **f**, **g** Representative photos and quantification of lung metastases in C57BL/6J mice. After 21 days, lungs (*n* = 7 for each group) were collected and fixed via vascular perfusion using PBS and formalin. **e** Numbers of tumour focuses and **f** the covered lung areas were measured using ImageJ software. (**P < 0.01, ***P < 0.005, ns non-significant).

The results clearly show that the deletion of the cleavage site and prevention of the CA IX ectodomain shedding led to increased growth of subcutaneously grafted primary tumours (no adverse effects were observed). The B16-NS-CA IX tumours showed both bigger volume and higher weight than the B16-FL-CA IX tumours (Fig. 6a, b), albeit both immunohistochemistry and western blotting analysis showed that B16-NS-CA IX tumours contained similar level of CA IX protein than B16-FL-CA IX tumours (Fig. 6c, d).

Finally, we performed an in vivo lung colonisation assay to evaluate the formation of experimental metastases.^44,45^ The B16-FL CA IX and B16-NS CA IX cells were intravenously inoculated into mice via tail veins (total 17 drug naïve animals in two experimental groups of seven and one control group of three), and allowed to colonise lungs and form metastatic lesions over 21 days (no adverse effects were observed). Then the mice were euthanised, the lungs were perfused with PBS, dissected and photographed.

Importantly, evaluation of the metastatic colonies (clearly visible due to the black colour of the B16 melanocytes) (Fig. 6g) showed that B16-NS-CA IX cells formed an increased number of lesions (albeit the difference was not statistically significant) (Fig. 6e) and the lesions covered a significantly larger lung area as compared to B16-FL-CA IX cells (Fig. 6f). Thus, the NS CA IX expressing cells appear to possess similar ability to survive in circulation and extravasate as the FL-CA IX cells, but higher competence to home and survive in lungs, and form larger metastatic lesions possibly due to increased intercellular adhesion, invasiveness, proliferation and/or ability to cope with microenvironmental conditions in the secondary site. These results are in agreement with the previous observation of larger B16-NS-CA IX primary xenografts (Fig. 6a, b) and suggest that the cell-tethered CA IX supports the pro-metastatic phenotype of cancer cells.

## Discussion

Up till now, virtually none of the published functional studies on CA IX have taken into consideration that its ectodomain can be cleaved and released to the extracellular space. However, in certain stressing physiological conditions, elevated shedding of the ectodomain can affect protective and pro-tumorigenic functions of the cell-membrane-bound CA IX molecules with possible consequences on cancer progression.^7,30^

In order to tackle the role of cell-associated CA IX in the absence of its ectodomain cleavage, we used an approach based on construction and ectopic expression of the non-shed (NS) CA IX mutant. This mutant was used as a model mimicking the physiological situation in tumours, in which shedding of CA IX might be hampered due to the absence of ADAM17, its inactivation by TIMPs-mediated inhibition, its aberrant maturation and activation, or due to somatic mutations affecting the CA IX folding and interaction with the proteinase.^46,47^

The impairment of CA IX shedding was accomplished by deletion of 10 amino acids from the stalk region linking the catalytic CA domain to the transmembrane region. The CA IX stalk consists of 22 amino acids and thus fulfils the size requirement formulated in the study on IL-6R shedding.^48^ It contains two putative ADAM17 cleavage sites, specifically A∗A at positions 400∗401 and P∗V at positions 403∗404 that both conform to predicted preferences at the cleavage positions P1 and P1′, whereas the surrounding positions are only partially preserved. Both basal and activated CA IX shedding was completely abolished by the deletion of amino acids 393–402 suggesting that the cleavage occurs between the amino acids at positions 400∗401. Thus, CA IX appears to contain a unique ADAM17 cleavage site SSPRA/AEPVQ that differs from the cleavage cites of pro-TNFα PLAQA/VRSSS, pro-TGFα ADLLA/VVAAS and IL-6 receptor SLPVQ/DSSSV.^48–50^

Despite the impairment of shedding, ADAM17 remains in close proximity to the NS-CA IX mutant, indicating that the cleavage site deletion does not perturb the ectodomain folding and allows for CA IX recognition by the proteinase. Moreover, increased PLA signal and elevation of ADAM17 protein in cells expressing NS-CA IX may suggest that the association with NS-CA IX could lead to ADAM17 stabilisation. This idea is in accord with previous observations that the cell-surface availability of ADAM17 can be controlled through multilevel regulation including maturation, trafficking, activation, endocytosis-recycling-exocytosis mechanisms, or by shedding of its ectodomain via other ADAMs.^46,47,51,52^ It is quite conceivable that ADAM17 remains sequestered by the substrate and dissociates only after the execution of cleavage. However, the CA IX cleavage actually cannot happen in the absence of the cleavage site presumably causing that the NS-CA IX variant keeps ADAM17 in the long-term assembly and thereby prevents its degradation and/or shedding.

At this point it is important to note that ADAM17 might not be the only executor of the CA IX ECD cleavage, because neither its inhibition nor its silencing is able to completely block the CA IX shedding. Remaining fraction of the CA IX ECD detectable in the culture medium supports an involvement of additional proteases, identity of which remains to be elucidated.

Further investigations of NS-CA IX showed that it retains the features of the FL-CA IX protein with respect to the ability to form oligomers, internalise, interact with bicarbonate transporters (represented here by AE2) and acidify extracellular pHe. This is not surprising as all these molecular aspects of CA IX depend on its transmembrane position as well as on the integrity of the extracellular domain and the intracellular tail that are apparently not affected by the cleavage site deletion.

On the other hand, biological effects of NS-CA IX diverge from those exerted by the cleavable full-length CA IX protein. This was already apparent in the altered levels of several extracellular proteins detected in culture media from C33a cells expressing NS-CA IX in comparison to cells with FL-CA IX. Basigin (elevated in NS-CA IX cells) is known to promote malignant development in many cancer types via stimulation of glycolytic metabolism.^53^ This molecule can be secreted or released from extracellular microvesicles and mediate tumour-stroma interactions leading to increased tumour growth, invasion, and metastasis.^54^ In contrast, thrombospondin-1 (reduced in NS-CA IX cells) is a key secreted anti-angiogenic factor, repression of which is essential for tumour growth.^55^ Interesting counter-alterations were observed with extracellular levels of insulin-like growth factor-binding proteins, namely IGFBP3 (down with NS) and IGFBP2 (up with NS), both in normoxia and hypoxia. IGFBP3 is known to have IGF-dependent and independent anti-proliferative and pro-apoptotic effects in many tumour cell types, while there is abundant evidence for growth-promoting effects of IGFBP2 in diverse cancer models.^56,57^ Although the functional attributes of these particular IGFBPs are cell type-dependent and often contradictory, they seem to correspond to both in vitro and in vivo observations made in the context of our study. Thus, the NS-CA IX associated extracellular changes are indicative of pro-tumorigenic signalling. These alterations can be faithfully correlated to the lack of the CA IX ectodomain cleavage, because we used the same cellular background and ectopic expression with comparable levels of FL-CA IX and NS-CA IX. The observation of IGFBP2/3 changes may be related to the earlier finding that cell-tethered CA IX can mediate activation of PI3K pathway via its intracellular tail, which can then affect levels of IGFBP2/3 proteins.^56,58,59^ It also cannot be excluded that IGFPB proteins can be affected indirectly by modified expression of THSB1 or basigin. Noteworthy, it was previously shown that CA IX expression in human bladder tumour cells negatively correlates with IGFBP3 mRNA, thus supporting the results of this study.^60^

In addition, the cells that express non-cleavable, cell-membrane-bound NS-CA IX display intrinsic cancer-promoting properties including higher migration capability and increased invasiveness than the cells that express cleavable FL-CA IX protein. Even more importantly, impairment of CA IX shedding results in improved primary tumour growth in vivo. Finally, the cells expressing the NS-CA IX mutant exhibit higher colonisation and metastatic growth propensity than the cells carrying FL-CA IX. These pro-metastatic effects can presumably reflect more invasive phenotype of NS-CA IX cells, their better cooperation though intercellular adhesion and subsequently, their improved local survival and growth in metastatic lesions (mirroring the situation in primary tumours).

Based on the current knowledge of CA IX regulation and functioning, we can propose two mutually non-exclusive explanations for these observations. First, cleavage of FL-CA IX releases the CA IX ectodomain to the extracellular space where it might interfere with the local function of the cell-associated CA IX protein e.g. by competitive interaction with CA IX partners in the plasma membrane or in the extracellular space. In accord with results of an earlier study showing a repellent effect of the soluble CA IX ectodomain, it may also destabilise cell–cell cohesion and/or cell-ECM adhesion that are critical for initial tumour growth and metastatic colonisation.^32^ Second, cell-membrane tethering of CA IX is required for proper execution of pH-regulatory and cell-ECM adhesion functions that protect tumour cells from microenvironmental stresses and contribute to their invasiveness and resistance to cytotoxic therapy. This is in line with the recently published observation that cancer cells with high plasma membrane CA IX signal are more viable and chemoresistant than the cells with low or no membrane CA IX signal despite being cultured together in the same dish and exposed to the same extracellular medium.^30^ This idea is also compatible with the signalling that can be emitted and accepted by the intracellular tail of the membrane-anchored CA IX in the form of PI3K pathway activation involved in the growth-promoting function of CA IX and/or PKA-mediated phosphorylation involved in the inside-out catalytic activation of CA IX.^12,58^

Thus, while previous studies showed that ectodomain shedding reduces the abundance of cell-associated CA IX especially in stressing conditions (such as chemotherapy^30^), here we provide the first evidence that it also affects the biological role of the cellular CA IX. We demonstrate that the impairment of ectodomain cleavage and expression of the non-shed CA IX mutant in cancer cells leads to increased invasiveness, primary tumour growth and metastatic colonisation. Our results indicate that shedding of the CA IX ectodomain is a negative control mechanism that can modulate the function of cellular CA IX at distinct steps of tumour development. However, in vitro and in vivo models used in this study address only selected events of the cancer progression scenario and thus, further investigations of CA IX shedding are underway to better elucidate this phenomenon.

The data presented here also support the view that CA IX shedding should be taken into account when attributing functions to cell-associated CA IX and when developing new CA IX-targeted therapeutic strategies. It was previously observed that chemotherapeutic drugs and also inhibitors of CA IX induce CA IX shedding and depletion of the target CA IX from the cell surface and that those cells that maintain CA IX on the plasma membrane, are more viable and resistant to treatment.^30^ The present study supports this finding and suggests that the outcome of chemotherapy (either with conventional drugs or with inhibitors) might be compromised in resistant cells with the cell-associated CA IX and indicate that additional strategies, e.g. combination with direct targeting of cells carrying CA IX via antibodies inducing cytotoxic effects might be needed to achieve better anti-cancer effect.

## Supplementary information

Supplemetary figures 1–6

## Data Availability

All data generated or analysed during this study are included in this published article (and its supplementary information files).
