# Supplementary material for: Impairment of carbonic anhydrase IX ectodomain cleavage reinforces tumorigenic and metastatic phenotype of cancer cells
Source: Br J Cancer. 2020 Mar 25;122(11):1590–603. doi: 10.1038/s41416-020-0804-z (PMC7250822; doi:10.1038/s41416-020-0804-z)
Supplement: Supplementary file 1 — Supplemetary figures 1–6 [file 41416_2020_804_MOESM1_ESM.pdf]

SUPPLEMENTARY FIGURES

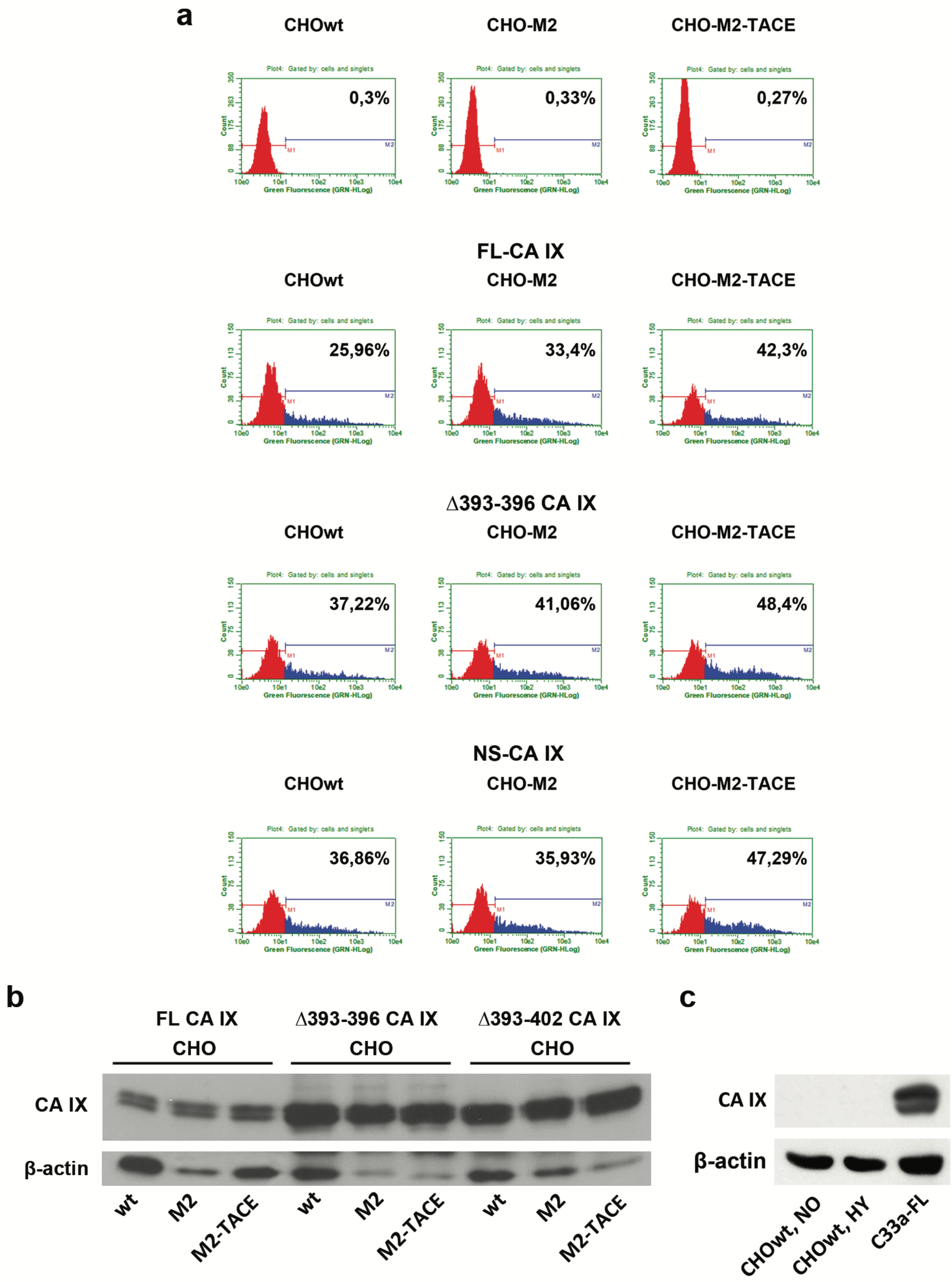

Supplementary figure 1. CA IX protein expression in transiently transfected CHO cells. (**a**) FACS analysis of CHO-wt, CHO-M2 and CHO-M2-TACE cells transiently transfected with the variants of the CA9 cDNA in pcDNA3.1+ plasmid as described in Materials and Methods. The living cells were stained with the CA IX-specific monoclonal antibody M75 and analysed on Guave EasyCyte plus (Millipore). The cells expressing CA IX are depicted in blue. (**b**) Cell lysates at volumes corresponding to those used for ELISA in Figure 1f, were analysed by Western blotting. Loading control represent β-actin of both transiently-transfected and non-transfected cells and therefore cannot be directly related to the levels of CA IX. (**c**) Western blotting analysis of control non-transfected CHO cells compared to FL-CA IX-transfected C33a cells confirms the absence of endogenous CA IX in CHO cells.

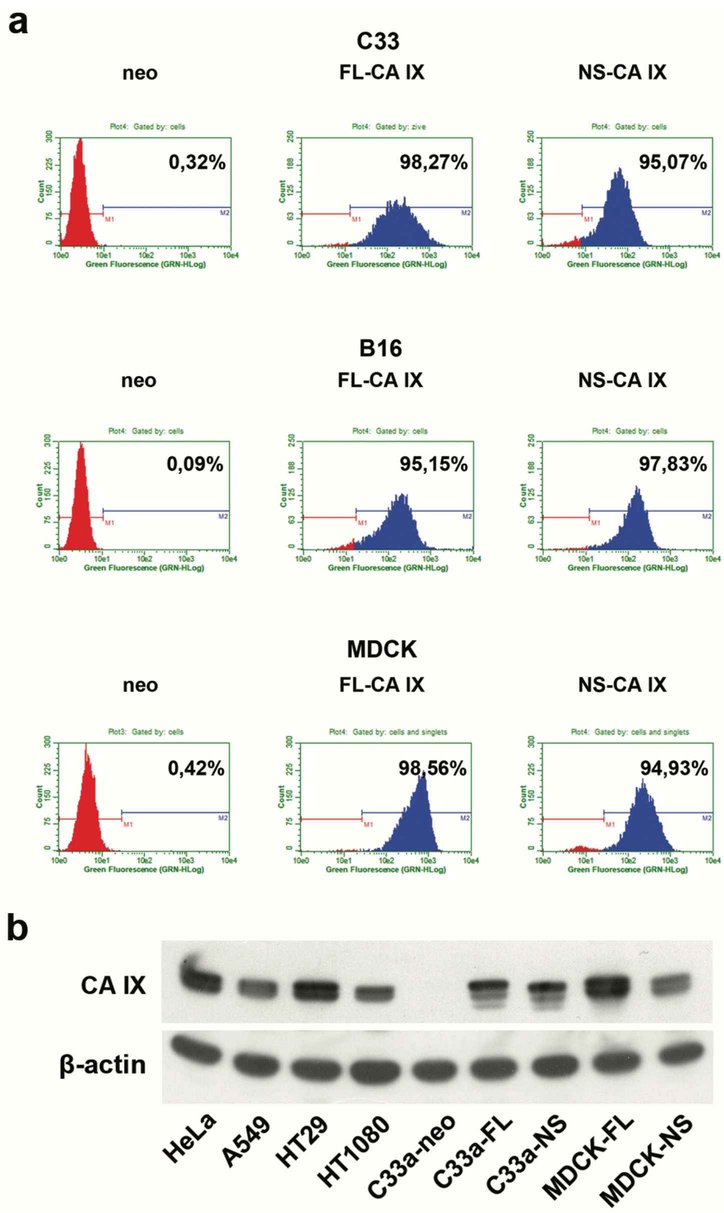

Supplementary figure 2. Endogenous versus ectopic CA IX protein levels in various cells lines. (a) Flow cytometric analysis of cell lines used in this study to compare endogenous and ectopic constitutive expression of CA IX. The cells were processed and data are presented as described in Suppl. Fig. 1a. (**b**) Human cancer cell lines (HeLa, A549, HT29, HT1080) endogenously expressing CA IX in response to hypoxia (2% O_2 for 24 h) compared to transfected C33a cells and MDCK cells. Western blotting analysis of cell lysates with the CA IX-specific M75 antibody and anti-β-actin antibody for the loading control. Results show that the transfected cells express CA IX at levels corresponding to natural conditions.

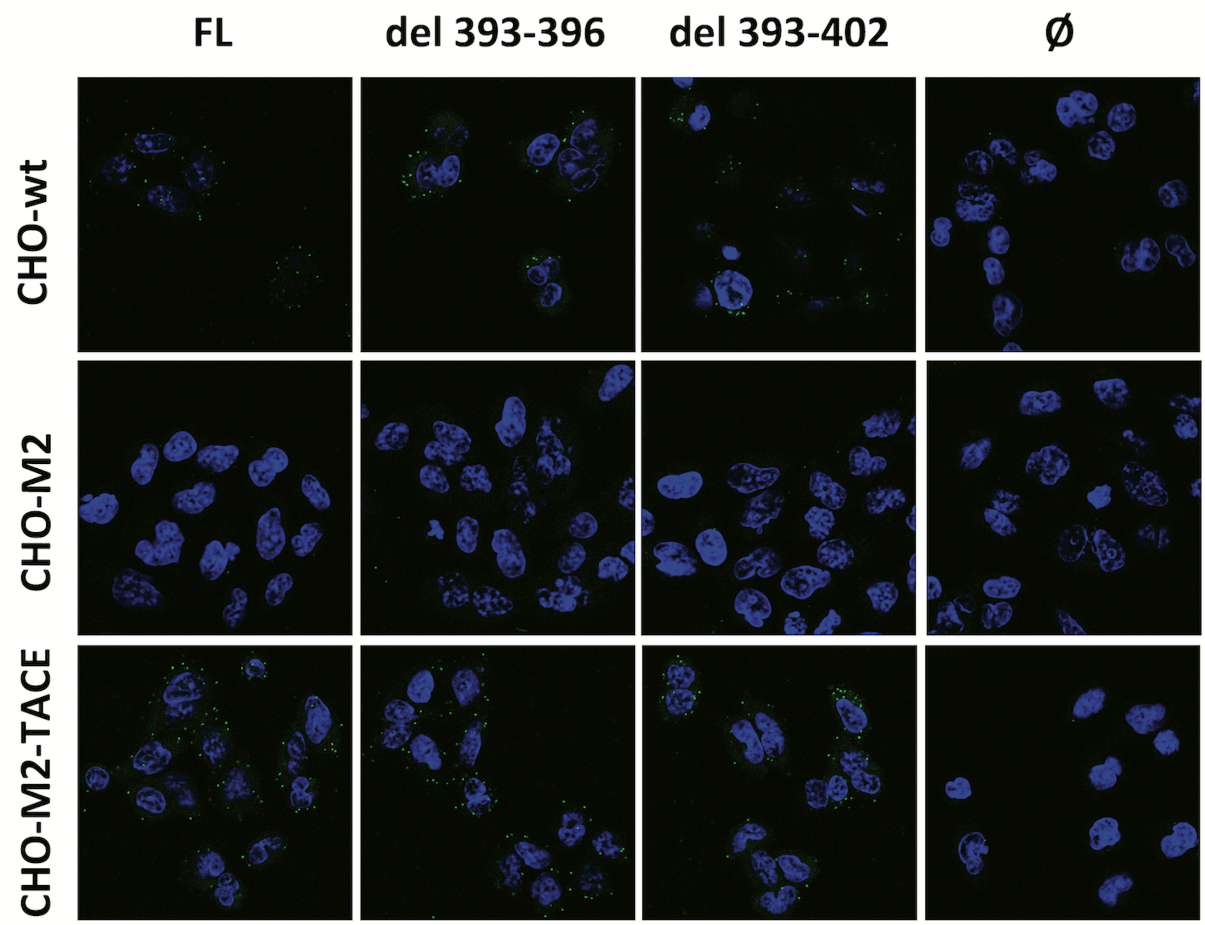

Supplementary figure 3. Interaction between CA IX and ADAM17 in variants of CHO cells with endogenous (CHO-wt), defective and ectopic ADAM17. Proximity ligation analysis of interaction between CA IX and ADAM17 in all three variants of CHO cells. The analysis was performed, evaluated and presented as described in Materials and Methods and as in Figure 2a.

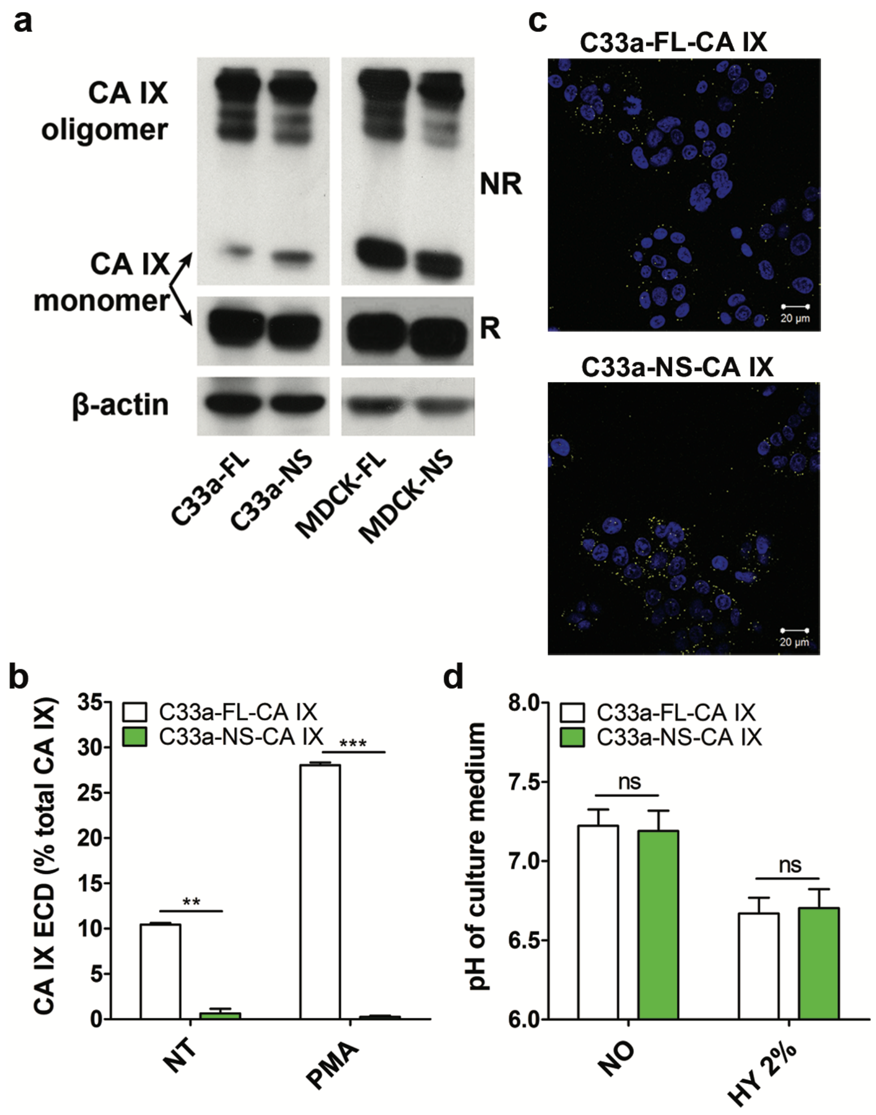

Supplementary figure 4. NS-CA IX variant forms oligomers and regulates pH similarly to FL-CA IX. a, Western blotting analysis of FL-CA IX and NS-CA IX variants in C33a and MDCK cells under reducing (R) and non-reducing (NR) conditions. **b,** ELISA evaluation of basal (NT – non-treated) and PMA activated ECD CA IX release from C33a-FL-CA IX and C33a-NS-CA IX cells was performed using V/10 antibody as a capture and mixture of biotinylated MAbs M75 and IV/18 as a detector. The graph illustrates percentage of ECD shed to medium from total cellular CA IX. Values represent average of CA IX levels measured in two experiments in triplicates. **c,** Monolayers of C33a-FL-CA IX and C33a-NS-CA IX cells subjected to PLA analysis. Both FL-CA IX and NS-CA IX interact with the anion exchanger AE2 (bicarbonate transporter) as visible from the yellow PLA signal **d,** Extracellular pH was measured in transfected C33a-FL-CA IX and C33a-NS-CA IX cells incubated for 48 h in normoxia (NO) and hypoxia (HY, 2% O_2), respectively. The graph shows pH values obtained with both variants that were able to acidify extracellular pH equally under hypoxic conditions. Data represent the mean ± SD of three independent experiments. Extracellular acidification reflects the enzymatic activity of both CA IX variants. (** P< 0.01, *** P<0.005, ns = non-significant)

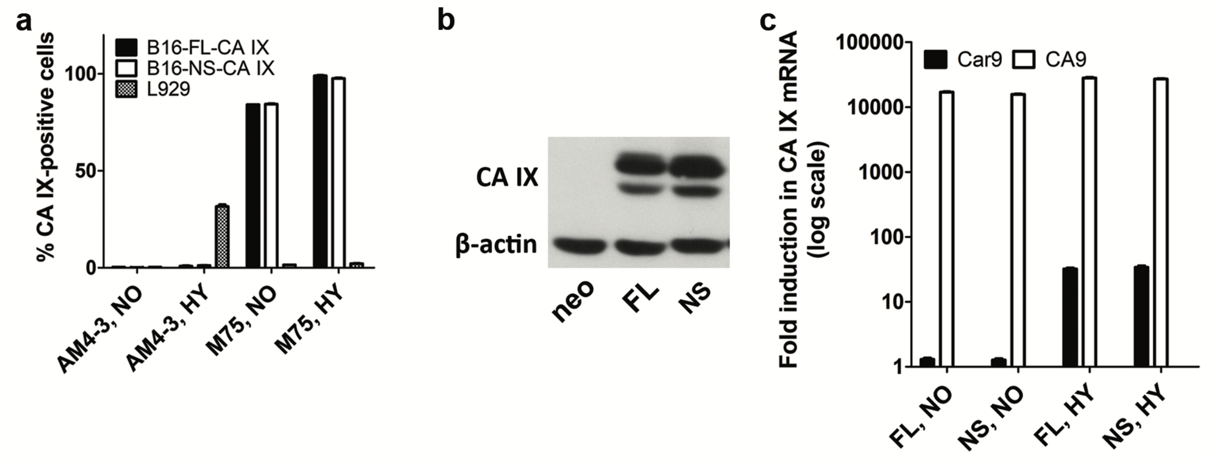

Supplementary figure 5. Expression of the endogenous mouse CA IX and transfected human CA IX proteins in B16 mouse melanoma cells. a, flow cytometric analysis: cell lines B16-FL-CA IX, B16-NS-CA IX and L929 (mouse cell line derived from normal subcutaneous tissue with endogenous expression of mCA IX) were cultured for 40 h in normoxic or hypoxic condition. The cells were trypsinized from the dishes into culture medium, centrifuged at low speed, washed twice with Versene, incubated for 1.5 h at 4 °C with the M75 or AM4-3 monoclonal antibody (5 µg/ml), centrifuged, washed twice with Versene and incubated with the secondary anti-mouse ALEXA Fluor® 488 antibody for 1 h at 4 °C. After washing with Versene, the analysis was performed on Guava EasyCyte plus (Merck Millipore, MA, USA) flow cytometer. Data were analysed with Guava Soft™ software version 5.2 (Merck Millipore, MA, USA). Using monoclonal antibodies AM4-3 (specific for the mouse CA IX) and M75 (specific for the human CA IX) shows that B16 cells express only negligible amount of the mouse CA IX protein, in contrast to L929 mouse fibroblasts. However, transfected B16 cells do express human FL-CA IX and NS-CA IX in comparable levels as confirmed also by **b,** Western blotting of FL-CA IX, NS-CA IX and mock-transfected B16-neo cells. **c,** qPCR analysis of the transfected B16 cells confirms the diminished expression of the mouse Car9 mRNA compared to the human CA9 mRNA. Following oligonucleotides were used: *Car9* sense: 5'-ATCACCCAGGCTCAGAACACAC-3' and antisense: 5'- TTCTTCCAAGTGGGACAGCAAC-3', *CA9* sense: 5'-TATCTGCACTCCTGCCCTCTG -3' and antisense: 5'-CACAGGGTGTCAGAGAGGGTG-3'. NO = normoxia, HY = hypoxia.

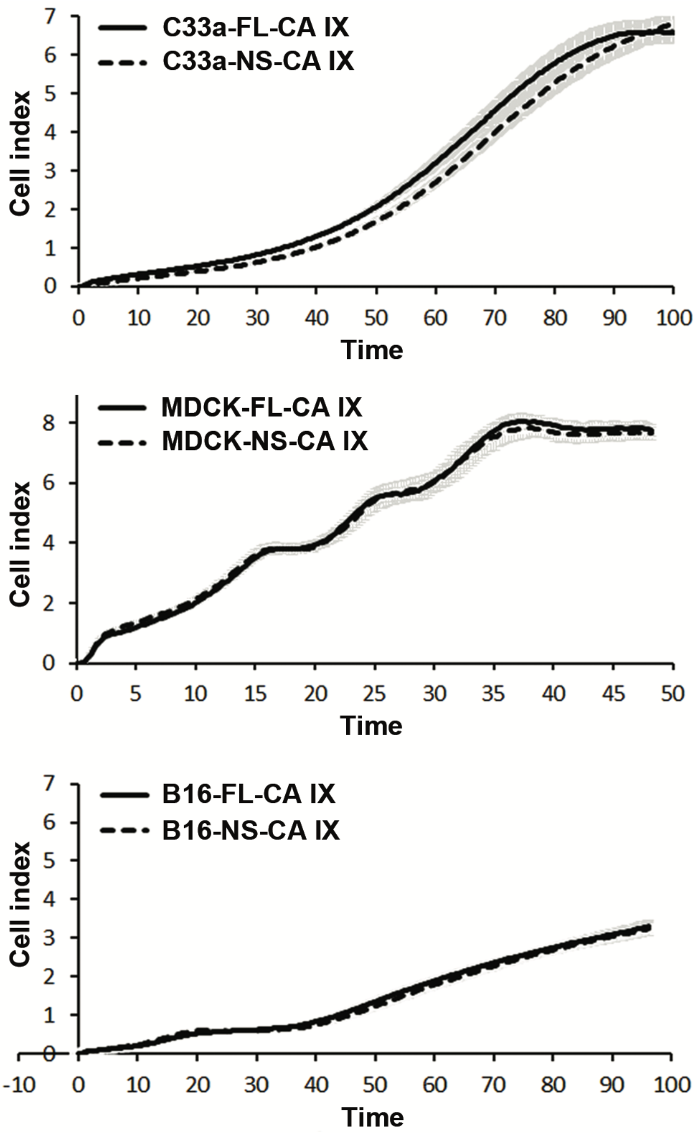

Supplementary figure 6. Cells expressing FL-CA IX versus NS-CA IX show similar proliferative capacity. Cell proliferation was analysed using E-plate 16 and the xCELLigence Real-Time Cell Analyzer as described in Materials and Methods. MDCK, C33a and B16 cells (5× 10^3 /well) transfected with FL-CA IX (full line) and NS-CA IX (dashed line) were added in quadruplicates to the wells of the E-plate. Proliferation was expressed as cell index representing relative change of impedance monitored every 15 min for 100 h.

Supplementary material. The ARRIVE Guidelines Checklist Animal Research: Reporting *In Vivo* Experiments
